# Endomembrane-Targeting *Plasmodiophora brassicae* Effectors Modulate PAMP Triggered Immune Responses in Plants

**DOI:** 10.3389/fmicb.2021.651279

**Published:** 2021-07-01

**Authors:** Md Musharaf Hossain, Edel Pérez-López, Christopher D. Todd, Yangdou Wei, Peta C. Bonham-Smith

**Affiliations:** ^1^Department of Biology, University of Saskatchewan, Saskatoon, SK, Canada; ^2^Department of Plant Sciences, Laval University, CRIV, Quebec City, QC, Canada

**Keywords:** *Plasmodiophora brassicae*, effectors, endomembrane, programmed cell death, pathogen-associated molecular pattern

## Abstract

*Plasmodiophora brassicae* is a devastating obligate, intracellular, biotrophic pathogen that causes clubroot disease in crucifer plants. Disease progression is regulated by effector proteins secreted by *P. brassicae*. Twelve *P. brassicae* putative effectors (*Pb*PEs), expressed at various stages of disease development [0, 2, 5, 7, 14, 21, and 28 days post inoculation (DPI)] in Arabidopsis and localizing to the plant endomembrane system, were studied for their roles in pathogenesis. Of the 12 *Pb*PEs, seven showed an inhibitory effect on programmed cell death (PCD) as triggered by the PCD inducers, *PiINF1* (*Phytophthora infestans* Infestin 1) and *PiNPP1* (*P. infestans* necrosis causing protein). Showing the strongest level of PCD suppression, *PbPE15*, a member of the 2-oxoglutarate (2OG) and Fe (II)-dependent oxygenase superfamily and with gene expression during later stages of infection, appears to have a role in tumorigenesis as well as defense signaling in plants. *PbPE13* produced an enhanced *PiINF1*-induced PCD response. Transient expression, in *Nicotiana benthamiana* leaves of these *Pb*PEs minus the signal peptide (SP) (^Δ*sp*^*Pb*PEGFPs), showed localization to the endomembrane system, targeting the endoplasmic reticulum (ER), Golgi bodies and nucleo-cytoplasm, suggesting roles in manipulating plant cell secretion and vesicle trafficking. ^Δ*sp*^*Pb*PE13GFP localized to plasma membrane (PM) lipid rafts with an association to plasmodesmata, suggesting a role at the cell-to-cell communication junction. Membrane relocalization of ^Δ*sp*^*Pb*PE13GFP, triggered by flagellin N-terminus of *Pseudomonas aeruginosa* (flg22 – known to elicit a PAMP triggered immune response in plants), supports its involvement in raft-mediated immune signaling. This study is an important step in deciphering *P. brassicae* effector roles in the disruption of plant immunity to clubroot disease.

## Introduction

*Plasmodiophora brassicae* is the intracellular obligate biotrophic plant pathogen responsible for clubroot disease in the Brassicaceae. The complex life cycle of *P. brassicae* can be divided into two infection stages: primary infection of a root hair resulting in secondary zoospore production and secondary infection of cortical tissues by secondary zoospores, leading to the production of resting spores and the characteristic swollen gall or club-shaped root of an infected plant (Rolfe et al., 2016). Secondary infection is crucial to the completion of the *P. brassicae* life cycle and the production of the next-generation of resting spores.

To facilitate the colonization of a plant root, *P. brassicae* secretes effector proteins to manipulate or interfere with the pathogen-induced host processes (Schwelm et al., 2015). Putative *P. brassicae* effector proteins, expressed during primary infection in canola as well as a secondary infection in Arabidopsis, have been identified through transcriptome analysis (Pérez-López et al., 2018, 2020; Chen et al., 2019). While the functional importance of many of these effectors remains unknown, a methyltransferase (*Pb*BSMT) that methylates salicylic acid (SA), thereby disrupting SA-induced host defense pathways and increasing host susceptibility to *P. brassicae* infection was recently characterized (Ludwig-Müller et al., 2015; Bulman et al., 2019). Further, more recent reports have identified a *P. brassicae* MAPKKK protein as an elicitor for the generation of ROS and hypersensitive response (HR)-like cell death after transient expression in *Nicotiana benthamiana* (Jin et al., 2020) and a *P. brassicae* cysteine protease inhibitor SSPbP53 that targets cruciferous papain-like cysteine proteases to manipulate plant immunity (Pérez-López et al., Unpublished results).

To restrict an infection, plants have developed intricate coordinated networks of defense responses comprised of, pathogen-associated molecular patterns (PAMP)-triggered immunity (PTI), which when triggered results in cell death via the production of reactive oxygen species (ROS) and other mechanisms, and effector-triggered immunity (ETI), that together form the base of stable and long term resistance to pathogens (Hammond-Kosack and Jones, 1996; Jones and Dangl, 2006). Resistance to *P. brassicae* was first demonstrated in the two Arabidopsis ecotypes, Tsu-0 and Ze-0, with both showing an incompatible interaction to *P. brassicae* pathotype-e, characterized by a HR and lignification of the cell wall but no characteristic gall structures (Fuchs and Sacristan, 1996). In the evolutionary pathogen–host arms race for compatible interaction, successful pathogens most often target and subvert the tightly interconnected pathways such as protein synthesis, endomembrane trafficking and cellular degradation (autophagy and proteasome-mediated degradation) inside a host cell (Langin et al., 2020). The host counters, starting right at the plasma membrane (PM) with immune receptor activation, vacuolar vesicle trafficking and membrane fusion at the PM (Chinchilla et al., 2006; Teh and Hofius, 2014), endocytic recycling (Chinchilla et al., 2006), secretory pathway defense response (Bartetzko et al., 2009) and endomembrane relocalization of host proteins between membrane compartments (Engelhardt et al., 2012). The endomembrane system is a complex intracellular membrane network comprised of the endoplasmic reticulum (ER), Golgi apparatus, endosomes, vacuoles and PM, all connected via vesicle transport, that plays an important role in cellular homeostasis and signal transduction in response to external stimuli. Endomembrane trafficking and its membrane compartment dynamics are pivotal to limiting pathogen spread within the host and in turn, are often targeted by pathogen effectors to subvert host immunity (Gu et al., 2017).

The importance of nanodomains within the PM in early defense signaling and cell to cell communication has been well-documented in plant cells (Raffaele et al., 2009; Perraki et al., 2014; Gronnier et al., 2017; Sasaki et al., 2018; Albers et al., 2019). However, while PM lipid rafts are important in the activation of the human immune system, several intracellular pathogenic bacteria can hijack these rafts to facilitate entry into the host cell or modulate defense signaling for survival inside the cell (Mañes et al., 2003; Hartlova et al., 2010). The lipid and protein composition of plant lipid rafts are similar between plants and reflect that of animal lipid rafts, suggesting similar functions to those of animal lipid rafts, e.g., signal transduction and cellular trafficking (Morel et al., 2006; Lefebvre et al., 2007). Moreover, it has also been suggested that lipid rafts in root cells may have a role in symbiotic infection in *Medicago truncatula* (Lefebvre et al., 2007).

From a cDNA library generated from canola galls, we have identified a number of *P. brassicae* effectors that, by localizing to different sub-compartments of the plant cell endomembrane system, as well as the manipulation of plant-triggered programmed cell death (PCD), suggest a role in a successful *P. brassicae* infection and colonization of the plant root.

## Materials and Methods

### Plant Materials and Growth Conditions

*Arabidopsis thaliana* Columbia-0 plants, for inoculation and expression analysis by RT-PCR, were grown in Sunshine Mix #3 soil (Sun Gro Horticulture Inc., Vancouver, BC, Canada) at 22°C, 16 h light/8 h dark and a light intensity of 100 μmol photons m^–2^ s^–1^ in a Conviron E8 growth chamber (CMP6050 control system). *N. benthamiana* seeds were sown on soil and stratified for 2 days at 4°C before transferring to similar growing conditions as above. Transplanted seedlings were grown in a growth chamber under 16 h light/8 h dark, 25°C and light intensity of 160 μmol photons m^–2^ s^–1^ controlled conditions. Transplantation of both *A. thaliana* and *N. benthamiana* seedlings was done 10 days after germination.

### Pathogen Materials, Inoculum Preparation, and Infection Assay

A single spore isolate of *P. brassicae* pathotype-3 (Strelkov et al., 2006), obtained from Dr. Gary Peng (AAFC, Saskatoon Research Centre), was propagated through *Brassica napus* cv. Westar (canola) plants. *P. brassicae* resting spores were extracted from 2 g of dry canola root galls by first submerging the gall in 0.25% Tween-20 solution for 5–7 min (Pérez-López et al., 2020). The gall was washed with 70% ethanol and twice with ddH_2_O, prior to grinding in a 10% sucrose solution using a mortar and pestle. The resulting suspension was passed through eight-layered cheesecloth and the filtrate was centrifuged at 100 rpm (Allegra 25R, Beckman Coulter Inc., Germany) for 5 min to remove root tissue debris. The supernatant was centrifuged at 2,500 rpm (Allegra 25R, Beckman Coulter Inc., Germany) for 5 min and the pellet, containing resting spores was washed twice with ddH_2_O before resuspension in 10 mL ddH_2_O. Resting spore concentration was determined using a hemocytometer and diluted to 4 × 10^7^ resting spores/mL. For infection studies, 14-day-old *A. thaliana* seedlings were inoculated at the soil level of the seedling stem with 500 μL of 4 × 10^7^ resting spores/mL. Control seedlings were treated with 500 uL of ddH_2_O. Each set of plants were grown on separate trays in the same growth chamber. Three independent biological replicates were carried out for both control and treated plants, with each experimental timepoint consisting of 10 or more plants.

### Putative *Plasmodiophora brassicae* Effectors and Their Functional Annotation

Sequences from a cDNA library from 35-day-old canola clubroot galls were screened for *P. brassicae* putative effectors (*Pb*PEs) using a bioinformatics pipeline (Supplementary Figure 1). Initial trimming of sequences was done using Phred (Ewing and Green, 1998; Ewing et al., 1998) with a quality threshold of 0.05. Vector sequences were identified by multiple sequence alignment using MUSCLE (EMBL-EBI) and removed using crossmatch^1^. Small (<75 bp) and duplicate sequences were removed using CD-HIT^2^ with a 97% identity cut-off. Non-redundant cDNA sequences were mapped against the *B. napus* and *P. brassicae* genomes using Spliced Transcripts Alignment to a Reference (STAR: Dobin et al., 2013). All sequences that mapped to the *P. brassicae* genome and those with no hits were translated to putative protein sequences using web server ExPASy tools^3^ and ORF finder^4^ and screened against the *P. brassicae* non-redundant proteome using BlastX (NCBI) with an E-value threshold of 0.001. All *P. brassicae* positive protein sequences were surveyed with the signal peptide (SP) prediction program SignalP v5.0 (organism group = Eukaryotes)^5^ with a D-cut-off score above or equal to 0.7 and those sequences with a predicted transmembrane domain (TMD) using TMHMM v2.0^6^ and Phobius^7^ and/or an ER retention signal motif (ScanProsite web server)^8^ were excluded from the final list of *Pb*PEs (Supplementary Table 1). Functional annotation of the final *Pb*PEs was carried out using HMMER^9^ and the rapid functional annotation server PANNZER^10^ with a *Z*-score threshold of 0.5. Blast2GO annotations based on functional descriptions with the top 20 hits were also considered and listed. *Pb*PE functional domains were identified using the Conserved Domain Database (CDD)^11^. The molecular weight, theoretical isoelectric point (pI) and amino acid length of *Pb*PEs were calculated using ProtParam^12^ and prediction of the subcellular localization of the *Pb*PEs was carried out using LOCALIZER^13^.

### Signal Peptide Validation Using a Yeast Signal Sequence Trap Assay

*Plasmodiophora brassicae* PE SP functionality was tested in yeast strain YTK12, which is deficient in growth on sucrose or raffinose medium without an active invertase secretory system (Oh et al., 2009). Coding sequences of *Pb*PE SPs were amplified from cDNA with the addition of 5′-*Eco*R1 and *Xho*I-3′ restriction sites and cloned, in frame with the SP-deficient invertase gene, into the pSUC2 vector. YTK12 was transformed with the resulting constructs using the Li-Acetate method (Gietz and Woods, 2002) and positive clones, selected on CMD-W media (Yu et al., 2017; Pérez-López et al., 2020), were confirmed by colony PCR. Positive yeast YTK12 transformants were grown on YPRAA selective media to select for invertase secretion (Yu et al., 2017; Pérez-López et al., 2020). For the TTC-(2,3,5-triphenyl tetrazolium chloride)-colorimetric assay, positive yeast YTK12 transformants were grown in YPD media for 36 h and pellets were collected from 1.5 mL cell suspension after centrifugation (Thermo Scientific Sorvall Legend Micro 21R) at 20,000 × *g* for 2 min. Pellets were washed twice with distilled water before re-suspending in 750 μl sterile distilled water. To this cell suspension 250 μl of 10 mM acetic acid–sodium acetate buffer (pH 4.7) and 500 μl 10% sucrose solution (w/v) was added and incubated at 37°C for 10 min. After centrifugation at 20,000 × *g* for 1 min, 100 μl of the supernatant was added to 900 μl of 0.1% TTC solution in a glass test tube and incubated at room temperature for 5 min. SP activity was investigated through secreted invertase reduction of the colorless 2,3,5-triphenyl tetrazolium chloride (TTC) to red-colored 1,3,5 triphenyl formazan (TPF) (Pérez-López et al., 2020). The previously identified Arabidopsis secretory protein, low molecular weight cysteine-rich 78 (*At*LCR78) (Shahzad et al., 2013; Pérez-López et al., 2020), was used as a positive control in both of these assays.

### RNA Extraction, cDNA Synthesis, and Semiquantitative RT-PCR Expression Analysis

Tissue samples from both *P. brassicae* inoculated and non-inoculated Arabidopsis roots at 0, 2, 5, 7, 14, 21, and 28-DPI, as well as resting spores from dry 35-day old canola galls, were collected in liquid nitrogen. Total RNA was extracted using the phenol-urea-LiCl method as previously described (Missihoun et al., 2011). RNA concentrations were measured using a Thermo NanoDrop 2000C spectrophotometer system (Thermo Fisher Scientific, Waltham, MA, United States). cDNA synthesis, for RT-PCR expression profiling, was carried out using the QuantiTect Reverse Transcription Kit (Qiagen, United States) using 200 ng total RNA. To avoid reaching saturation, semi-quantitative PCR was carried out using a low number of PCR cycles (*n* = 28). Semiquantitative RT-PCR expression data were generated from unsaturated gel image analysis using VisionWorks LS software^14^. Relative expression profiles of the *Pb*PEs were measured against the *P. brassicae* internal control *PbRPS17* (AF539801).

### Vector Construction and Subcellular Localization of *Pb*PEs

Predicted SP sequences were removed by amplification using appropriate paired AttB1 recombination cloning primers; 3′ to the SP sequence and the 3′ end of the *Pb*PE sequence. After removal of the SP sequence, *in planta* subcellular localization was determined for each ^Δ*sp*^*Pb*PE. ^Δ*sp*^*PbPE* cDNA sequences or cellular marker gene sequences were cloned into plant expression binary vectors with (pH7**X**WG2) or without (pH7WG2) fluorescent tags, using Gateway cloning technology (Thermo Fisher Scientific; Karimi et al., 2002). The cDNA sequence of each ^Δ*sp*^*PbPE*, sandwiched between attB1 and attB2 recombination sites, was inserted into the entry vector pDONR221/207/Zeo via a BP reaction. From there the ^Δ*sp*^*PbPE* sequence was added to the C-terminal *GFP* tagged binary vector pH7**F**WG2, via an LR reaction, with expression driven by the CaMV 35S promoter.

The mCherry-tagged sub-cellular marker gene constructs, in the pBIN20 binary vector backbone, were purchased from the Arabidopsis Biological Resource Centre^15^. The *GUS* expression construct, pH7WG2-*GUS*, was created from pENTR-*GUS* provided in the gateway cloning kit, with the *GUS* sequence inserted into pH7WG2 via an LR reaction. The *A. thaliana REMORIN 1.3* (AT2G45820.1) sequence was cloned into pH7**R**WG2, with a C-terminal *mRFP* fluorescence tag, for co-localization studies. A *GFP* construct, pH7WG2-*GFP*, was also generated for use as a negative control for the cell death assay and transient localization studies. All constructs were used to transform *Agrobacterium tumefaciens*, with positive transformants selected on LB medium supplemented with spectinomycin (100 mg/L), kanamycin (50 mg/L), or rifampicin (50 mg/L) and subsequently used to transform *N. benthamiana* for transient expression studies. All the constructs used and generated in this study are provided in Supplementary Table 2.

Subcellular localization of *Pb*PEs was determined by transiently expressing the ^Δ*sp*^*Pb*PE-GFP gene fusion-constructs, in *A. tumefaciens* at a final OD_600_ of 0.3, together with organelle-specific markers, in *N. benthamiana* leaves. Subcellular localization of the PEs was recorded 2-3 days after agroinfiltration. The localization of each ^Δ*sp*^*Pb*PE-GFP was visualized with a LSM880 inverted confocal laser scanning microscope (Zeiss, MN, United States) using a 40X water objective at GFP-required wavelengths. GFP and chloroplast autofluorescence was monitored using an Argon laser at 488/500–530 and 488/580–620 nm excitation/emission wavelengths, respectively. The mRFP and mCherry fluorescence tags were monitored using a Helium-Neon laser at 561/600 and 561/630 nm, excitation/emission wavelengths, respectively.

To classify the localization of ^Δ*sp*^*Pb*PE-GFPs at the cell periphery, *N. benthamiana* leaf segments (leaves) were plasmolyzed in 0.85 M KCl for 15 min before observation under the Zeiss LSM880 microscope using a 40X water objective as outlined above. Flg22 treatment was performed on leaves 2 days post infiltration and confocal images were taken 1 h after flg22 treatment.

Z-stack and time-lapse images were captured to provide further insight into the fluorescence distribution, association and dynamics of ^Δ*sp*^*Pb*PE localization in *N. benthamiana* leaf epidermal cells. To verify the localization profile for each ^Δ*sp*^*Pb*PE, multiple images were captured from different fluorescence-expressing cells. To avoid overexpression artifacts, transiently expressing cells, with comparatively low fluorescent signals, were imaged for analysis using FIJI ImageJ^16^. Fluorescence intensity plots were graphed based on the quantitative data measured in arbitrary units (a.u.), obtained from the region of interest of a confocal colocalized image represented by a blue line, using ImageJ. Each fluorescence channel in a colocalized confocal image represents the individual line graph in a fluorescence intensity plot.

### Agrobacterium Co-infiltration and Cell Death Assay

Screening of *Pb*PEs for cell death regulation was carried out, with a PCD assay using the inducers *PiINF1* elicitin and *PiNPP1* from *Phytophthora infestans*, as previously described (Kelley et al., 2010). ^Δ*sp*^*Pb*PEs were cloned under the control of the CaMV 35S promoter, using gateway cloning, as described above. Transient expression of ^Δ*sp*^*PbPE*s in *N. benthamiana* leaves was carried out as described in Sparkes et al. (2006). The third and fourth healthy leaves of 5-week-old *N. benthamiana* plants were infiltrated on the abaxial side with *A. tumefaciens* GV3101 (pMP90) strain carrying an inducer of PCD, pGR106-*PiNPP1* or pGR106-*INF1* plus a ^Δ*sp*^*PbPE* construct. pH7WG2-*GFP* was used as the negative control and pART-*PiSNE1* (suppressor of necrosis 1) as the positive control for the PCD regulation assays (Kelley et al., 2010).

The PCD assays were carried out using two different methods: (i) a single infiltration of an equal concentration (OD_600_ of 0.3 for each construct) mixed solution of inducer and ^Δ*sp*^*PbPE* and (ii) overlapping additions of inducer and ^Δ*sp*^*PbPE*, where leaves were first infiltrated with the ^Δ*sp*^*PbPE* 1 day before infiltration with the PCD inducer at a separate, but partially overlapping location, on the same leaf. The overlapping zone of infiltration was the area of co-expression and possible PCD suppression. Suppression or induction of PCD was monitored 5 days post infiltration with *PiINF1* and 7 days post infiltration with *PiNPP1*. The HR index was calculated from the mean percentage necrotic area per total infiltrated area on the leaves using the color threshold for FIJI ImageJ (see text footnote 16). Student’s *t*-test was conducted to identify statistically significant differences in co-infiltration treatment between *GFP* and *PbPE*s with PCD inducers at *p* = 0.01 and 0.05.

## Results

### Selection of *Plasmodiophora brassicae* Candidate Effectors

cDNAs from a full-length cDNA library of total RNAs extracted from *P. brassicae*-infected canola galls at 35 DPI with *P. brassicae* resting spores were sequenced and screened for *Pb*PEs. A total of 117 putative secretory protein (effectors)-coding cDNAs (PEs), comprising proteins with an N-terminal SP for secretion out of the pathogen into the plant cell (Supplementary Table 1), a transmembrane domain (TMD) and in some PEs an ER retention signal (HDEL, KDEL) at the C-terminus, were identified (Supplementary Figure 1). Removal of putative membrane located (TMD) proteins and proteins with ER retention signals, resulted in a final list of 52 *Pb*PEs (Supplementary Table 1), representing 44% of the total *P. brassicae* secretome identified from *in silico* study at the clubroot gall stage of infection in canola. Based on previously published RNA-seq data (Irani et al., 2018; Pérez-López et al., 2020), functional annotation and preliminary expression data, suggesting likely importance in pathogenesis, 15 *Pb*PE sequences were selected for further study.

**FIGURE 1:**
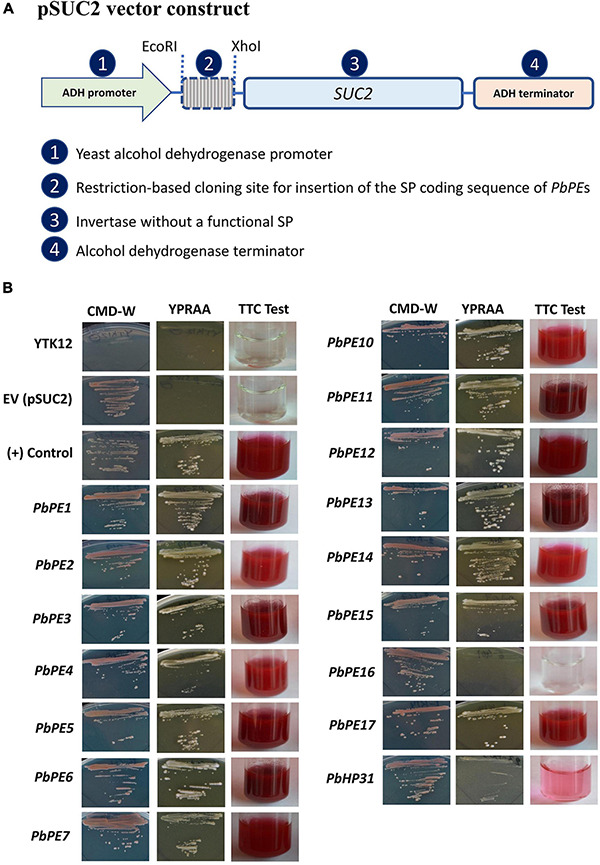
Functional validation of the *Plasmodiophora brassicae* PE signal peptides with a yeast secretion system. **(A)** pSUC2 vector construct containing the signal peptide sequences of the *P. brassicae* PEs cloned in frame with the invertase gene. **(B)** The invertase negative yeast strain (YTK12) transformed with pSUC2 constructs with a functional signal peptide grew on YPRAA selective media and invertase activity was visualized through reduction of TTC to the red–colored formazan. Intermediate color (pink) was also observed in the TTC test and considered negative in the validation of functional SP. Untransformed YTK12 and YTK12 transformed with empty vector (pSUC2) were negative controls and Arabidopsis secretory protein low molecular weight cysteine-rich 78 (*At*LCR78) was the positive control. These images are representative of three independent biological replicates.

Each of the 15 *Pb*PEs contained a predicted N-terminal SP (SignalP v5.0). Using a yeast secretion system (Gietz and Woods, 2002; Oh et al., 2009), SP functionality was established for 14 of the 15 *Pb*PE SPs, with the predicted *Pb*PE16 SP lacking function in both the growth and secretion (red) aspects of the assay (Figure 1). The subcellular membrane localization, in plants, of the 14 *Pb*PEs with functional SPs, was determined.

### *Pb*PEs Targeting to the Endomembrane System of the Plant Cell

To mimic secretion (i.e., processing and cleavage of the *Pb*PE SP during secretion) from the pathogen into the plant cell, each of the 14 *Pb*PE genes was cloned, minus the SP (Δsp), in frame with a green fluorescent protein (GFP) sequence, under the control of a single CaMV 35S promoter. Each of the resulting 14 ^Δ*sp*^*Pb*PE-GFPs was transiently expressed in *N. benthamiana* leaf epidermal cells.

**FIGURE 2:**
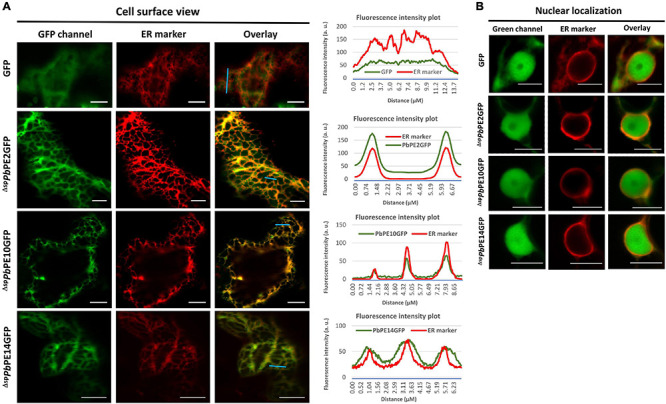
Endomembrane localization of *Pb*PEGFPs upon transient expression in leaf epidermal cells of *N. benthamiana*. Representative confocal micrographs showing free GFP and nuclear-ER localization of ^Δ*sp*^*Pb*PE2GFP, ^Δ*sp*^*Pb*PE10GFP and ^Δ*sp*^*Pb*PE14GFP in *N. benthamiana* leaves. **(A)** Red fluorescence corresponding to the ER marker, mCherry-HDEL (CD3-959) shows the cortical ER pattern and the right panels indicate the merge of green and red fluorescence channels. **(B)** Confocal images show nuclear and perinuclear localizations of the free GFP and GFP-tagged ^Δ*sp*^*Pb*PEs and mCherry-tagged ER marker, respectively. Fluorescence intensity plots show the representative localization patterns of the FP-tagged proteins along the light blue lines on image overlays. Scale bars = 10 μm.

**FIGURE 3:**
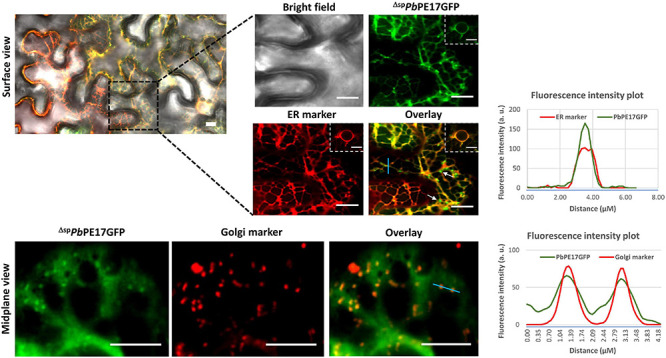
Endomembrane localization of *Pb*PE17GFP upon transient expression in leaf epidermal cells of *N. benthamiana*. ^Δ*sp*^*Pb*PE17GFP localizes in both ER and Golgi stacks (white arrows) but is excluded from nuclei upon transient expression in *N. benthamiana*. The concentrated green fluorescence on the face of cortical ER (white arrows) was identified as Golgi stacks by co-localization with the Golgi marker. Surface and mid-plane views of the cell show cortical ER and Golgi localization of the effector, respectively. Black dotted line highlights the zoom in version of the surface images. Inset images showing perinuclear ER localization of ^Δ*sp*^*Pb*PE17GFP in transiently expressed cells. Representative fluorescence intensity plots of co-localization of ^Δ*sp*^*Pb*PE17GFP with ER or Golgi markers are shown along the light blue line on image overlays. Scale bars = 10 μm.

**FIGURE 4:**
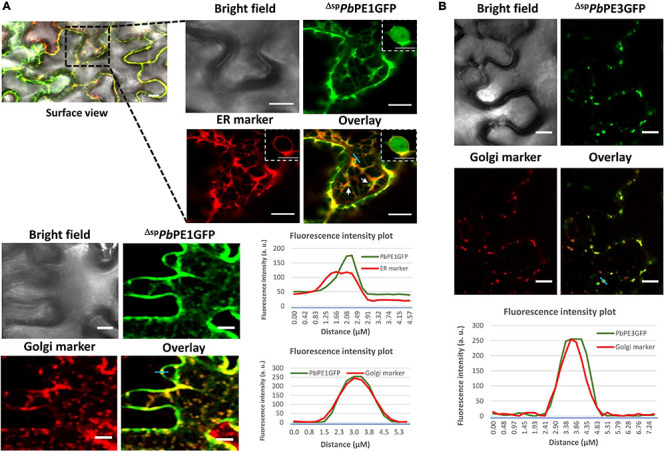
Endomembrane localization of *Pb*PE1GFP and *Pb*PE3GFP upon transient expression in leaf epidermal cells of *N. benthamiana*. **(A)** ^Δ*sp*^*Pb*PE1GFP co-localizes with ER and Golgi markers in *N. benthamiana* leaves. Nuclear localization of ^Δ*sp*^*Pb*PE1GFP was also shown in the inset picture. The concentrated green fluorescence on the face of cortical ER (white arrows) was identified as Golgi stacks by co-localization with the Golgi marker. **(B)** ^Δ*sp*^*Pb*PE3GFP co-localizes with the cis-faced Golgi stack marker, *Gm*Man^11– 49*aa*^-mCherry (CD3-968). Representative fluorescence intensity plots of co-localization of ^Δ*sp*^*Pb*PE1GFP with ER or Golgi markers and ^Δ*sp*^*Pb*PE3GFP with Golgi markers are shown along the light blue line on image overlays. Black dotted line highlights the zoom in version of the surface images. Scale bars = 10 μm.

Of the 14 ^Δ*sp*^*Pb*PE-GFPs, 12 localized to the endomembrane system, with localization to the ER being most prominent. ^Δ*sp*^*Pb*PE-GFPs; ^Δ*sp*^*Pb*PE2GFP, ^Δ*sp*^*Pb*PE10GFP and ^Δ*sp*^*Pb*PE14GFP all localized to the ER and the nucleus in *N. benthamiana* (Figure 2), whereas ^Δ*sp*^*Pb*PE17GFP, containing three ANK repeats and a predicted BTB domain, localized to both ER and Golgi bodies (Figure 3 and Supplementary Files 1, 2). The ER-mCherry (CD3-959) contains the ER retention signal (HDEL) at the C-terminus was used as a marker for co-localization studies. ^Δ*sp*^*Pb*PE1GFP, also containing three ANK repeats, localized to the ER, Golgi bodies and the nucleus (Figure 4A). ^Δ*sp*^*Pb*PE3GFP localized only to small, mobile, punctate structures, in the plant cytoplasm, that were identified as Golgi bodies after colocalization with the cis-faced Golgi stack marker, *Gm*Man^11–49*aa*^-mCherry (CD3-968) (Figure 4B), while ^Δ*sp*^*Pb*PE5GFP, ^Δ*sp*^*Pb*PE6GFP, ^Δ*sp*^*Pb*PE11GFP, and ^Δ*sp*^*Pb*PE12GFP all showed nucleo-cytoplasmic localization with ER fractions in the cell (Supplementary Figure 2). While the possibility cannot be ignored that nuclear localization was the result of diffusion, as seen for GFP alone (Figure 2B), if this was the case then with all selected *Pb*PEs being small, secreted protein-GFPs, one would expect that all *Pb*PE-GFPs would be found in the nucleus.

### ^Δ*sp*^*Pb*PE13GFP Localizes to PM Lipid Rafts

**FIGURE 5:**
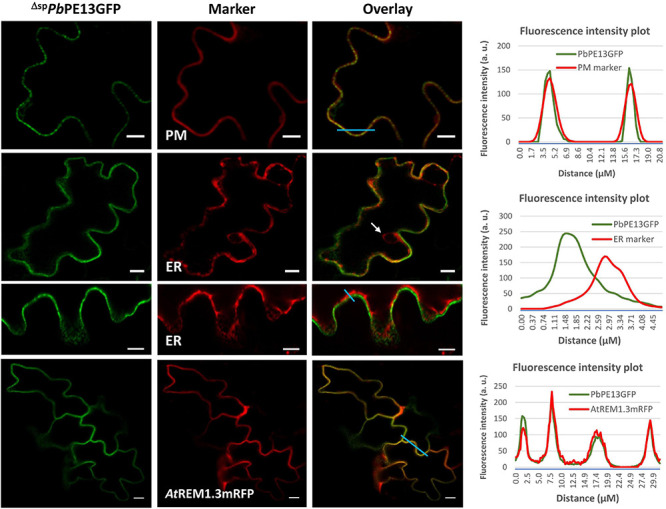
Localization of ^Δ*sp*^*Pb*PE13GFP to plasma membrane (PM) lipid rafts in *N. benthamiana* leaf cells. PM localization of ^Δ*sp*^*Pb*PE13GFP, together with punctate structures at the cell peri2phery. Co-localization images of ^Δ*sp*^*Pb*PE13GFP and a known PM-marker, mCherry-tagged Arabidopsis aquaporin *At*PIP2A (CD3-1007). ^Δ*sp*^*Pb*PE13GFP did not colocalize with perinuclear ER (white arrow). Co-localization of ^Δ*sp*^*Pb*PE13GFP with Arabidopsis REMORIN1.3mRFP in PM lipid rafts. Representative fluorescence intensity plots of co-localization of ^Δ*sp*^*Pb*PE13GFP with PM or ER or Remorin markers are shown along the light blue line on image overlays. Scale bars = 10 μm.

**FIGURE 6:**
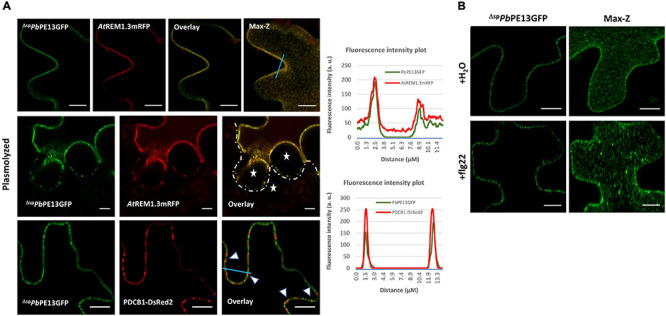
Association of ^Δ*sp*^*Pb*PE13GFP with plasmodesmata (PD) after transient expression in *N. benthamiana* leaf cells. **(A)** Close up images and maximum Z-projection of the co-localization of ^Δ*sp*^*Pb*PE13GFP and *At*REM1.3mRFP in punctate plasma membrane (PM) structures. Plasmolysis of the co-localized cells shows some retention of fluorescence signal at the cell wall; white stars indicate apoplastic spaces between adjoining cells. Co-localization of ^Δ*sp*^*Pb*PE13GFP with plasmodesmata associated protein PDCB1-DsRed2 at punctate PM structures (white arrowheads). **(B)** Re-organization of membrane localization of ^Δ*sp*^*Pb*PE13GFP upon flg22 treatment in *N. benthamiana* leaf cells. Confocal images of surface localization patterns of the effector were monitored upon flg22 treatment and compared with a H_2_O control. PM localization of ^Δ*sp*^*Pb*PE13GFP, together with punctate structures at the cell periphery using H_2_O control. flg22 treatment of the ^Δ*sp*^*Pb*PE13GFP transiently expressed leaves shows relocation of fluorescence signal into concentrated and mobile punctate structures as observed in the maximum-Z projection. Representative fluorescence intensity plots of co-localization of ^Δ*sp*^*Pb*PE13GFP with Remorin or PDCB1-DsRed2 markers are shown along the light blue line on image overlays. Scale bars = 10 μm.

*Pb*PE13 is a small hypothetical protein (143 amino acids) of unknown function that is not annotated as a *P. brassicae* protein in the NCBI database. ^Δ*sp*^*Pb*PE13GFP localized to punctate structures at the cell periphery that co-localized with PM intrinsic protein 2A (*At*PIP2A-mCherry CD3-1007) (Figure 5). Co-localization of ^Δ*sp*^*Pb*PE13GFP with the ER marker showed limited ER or perinuclear ER association (Figure 5), however, the punctate arrangements of ^Δ*sp*^*Pb*PE13GFP at the cell periphery co-localized perfectly with a PM lipid raft marker *At*REMORIN 1.3 (*At*REM1.3) tagged with C-terminal mRFP (Figures 5, 6A). To evaluate the robustness of the PM localization, ^Δ*sp*^*Pb*PE13GFP and *At*REM1.3mRFP co-expressing cells were plasmolyzed, resulting in a large fraction of the co-localized signal remaining in the retracting PM, with a small amount retained at the cell wall (Figure 6A). Co-expression of ^Δ*sp*^*Pb*PE13GFP with the known PD marker, plasmodesmata localized callose binding protein 1 (PDCB1-DsRed2) showed co-localization of some of the ^Δ*sp*^*Pb*PE13GFP punctate structures with PDCB1-DsRed2 signals (Figure 6A), suggesting that ^Δ*sp*^*Pb*PE13GFP associates with plasmodesmata.

*Plasmodiophora brassicae* primary and secondary zoospores are bi-flagellated, providing motility and facilitating attachment and infection of host cells. To avoid PTI and establish colonization in host plants, *P. brassicae* must regulate the flg22-FLS2 triggered innate immune responses during infection. Therefore, we investigated the dynamics of ^Δ*sp*^*Pb*PE13GFP localization upon flg22 perception in *N. benthamiana*. Treatment with flg22, a peptide derived from the flagellin N-terminus of *Pseudomonas aeruginosa*, triggered the association of *Nb*REM4 and fluorescence distribution due to membrane raft re-organization and compartmentalization within PM lipid rafts (Keinath et al., 2010; Albers et al., 2019). With a concentrated localization of ^Δ*sp*^*Pb*PE13GFP to PM lipid rafts, we wanted to determine if ^Δ*sp*^*Pb*PE13GFP showed a similar localization pattern to *Nb*REM4 upon biotic stress. To do so, we investigated the dynamics of ^Δ*sp*^*Pb*PE13GFP localization upon flg22 perception in *N. benthamiana* leaf epidermal cells. Maximum-Z projections show concentrated punctate structures due to the compartmentalization of ^Δ*sp*^*Pb*PE13GFP fluorescence in nanodomains of the PM after flg22 treatment (Figure 6B), suggesting that ^Δ*sp*^*Pb*PE13GFP can re-organize PM lipid rafts upon flg22 perception at the PM and may have a regulatory role in flg22/FLS2 triggered endocytosis and PTI response in plants.

### *Pb*PEs Targeting the Plant Cell Endomembrane System Are Differentially Expressed During Primary and Secondary Infection of Arabidopsis

**FIGURE 7:**
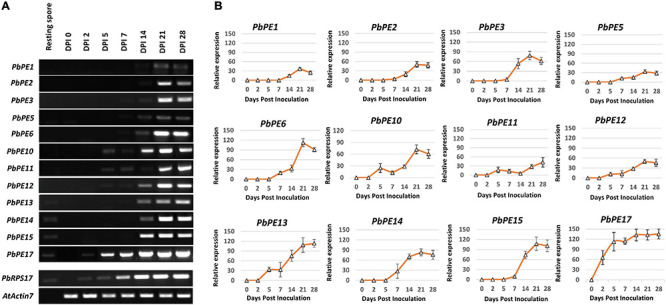
Gene expression profiles of plant endomembrane-localized *P. brassicae* PEs in the root sample of Arabidopsis plants infected with *P. brassicae* pathotype-3. **(A)** Semi-quantitative RT-PCR gene expression patterns of *P. brassicae* PE candidates at different time points; resting spore, 0, 2, 5, 7, 14, 21, and 28-days post inoculation (DPI). *P. brassicae* internal control, *PbRPS17* (AF539801) and Arabidopsis positive control, *AtACTIN7* (At5g09810). **(B)** Mean (±SD) relative expression profiles of candidate *P. brassicae* PEs. The relative expression of all *P. brassicae* genes was determined with respect to that of *PbRPS17* set at 100. *n* = 3 independent biological replicates.

Transcript levels for the 12 endomembrane-localizing *PbPE* genes were determined at 0, 2, 5, 7, 14, 21, and 28 DPI of Arabidopsis with *P. brassicae* pathotype-3 (Figure 7). Of the 12 *PbPE* genes, transcripts for seven (*PbPE1* to *PbPE11*) were not found in resting spores (Figure 7A) but were found at various stages of infection, during primary infection, after 2 days – *PbPE10*, *PbPE11* or after 5 days – *PbPE5*, *PbPE6*; or during secondary infection, after 7 days – *PbPE1*, *PbPE2*, *PbPE3.* Once initiated, all 12 *PbPE* genes showed continuous and increasing transcript levels up to 21 DPI, after which most showed decreased levels. Transcript for remorin-associated *PbPE13* was initially identified during primary infection, at 5 DPI, with no increase between 5 and 7 DPI, before peaking at 21 DPI (Figure 7). *PbPE10* was the only *Pb*PE to show a bimodal transcript level; increased transcript at 5 DPI, during primary infection and again at 21 DPI, during late secondary infection (Figure 7).

### *Pb*PEs Regulate the PTI Response in Plants

**FIGURE 8:**
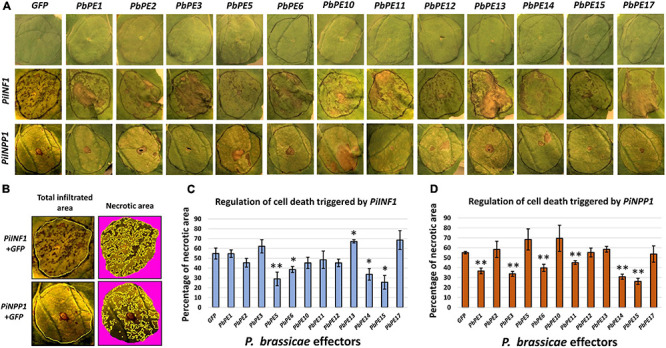
Endomembrane-localized *P. brassicae* PEs regulate cell death triggered by cell death inducers after transient expression in *N. benthamiana*. *Agrobacterium* strains carrying *P. brassicae* PE gene-vector constructs were co-infiltrated with or without cell death inducers, into leaves of *N. benthamiana*, to determine their effect on the regulation of cell death. *GFP* and cell death inducers (*PiINF1* and *PiNPP1*) were used as negative and positive controls, respectively. **(A)** Visual observation of *N. benthamiana* leaves infiltrated with *P. brassicae* effectors and cell death inducers. Infiltration with *GFP* or *P. brassicae* PEs alone did not result in cell death (1st row). Cell death phenotypes were scored 5 (*PiINF1*) and 7 (*PiNPP1*) days post infiltration after co-infiltration with *P. brassicae* PEs. **(B)** Quantitative measurement of cell death in necrotic areas, within the total infiltrated areas on the leaves, was calculated with ImageJ utilizing color threshold measures. Yellow lines indicate the selected regions for calculating total infiltrated areas and necrotic areas on the leaves. Mean percentage of necrotic areas of leaves co-infiltrated with **(C)** *PiINF1* or **(D)** *PiNPP1* and *P. brassicae* PEs. Each column represents a quantitative measure of the HR index in relation to the mean percentage of the necrotic areas of leaves and standard deviation for an effector of *P. brassicae*. *P-value < 0.05 and **P-value < 0.01. *Pi*INF1 = infestin 1 elicitin of *P. infestens*, *Pi*NPP1 = necrosis causing protein of *P. infestens. N* = three independent biological replicates.

The effect of the 12 secreted endomembrane targeting *P. brassicae* PEs on the plant PTI response was assessed using a cell death assay with *PiINF1* (*P. infestans* Infestin 1) and *PiNPP1* (*P. infestans* necrosis causing protein 1) as inducers of PCD (Figure 8). Agroinfiltration of *PiNPP1* or *PiINF1* with a *GFP* control construct induced PCD of *N. benthamiana* leaf cells (Figure 8A). *PiINF1* is a more potent inducer of PCD, with necrotic lesions prominent 5 days post infiltration with *PiINF1* + *GFP*, compared to *PiNPP1*, with necrotic lesions visible 7 days post infiltration with *PiNPP1* + *GFP*. The induction or suppression of PCD by each *Pb*PE was measured by the mean percentage of necrotic area within the infiltrated zone (Figure 8B).

**FIGURE 9:**
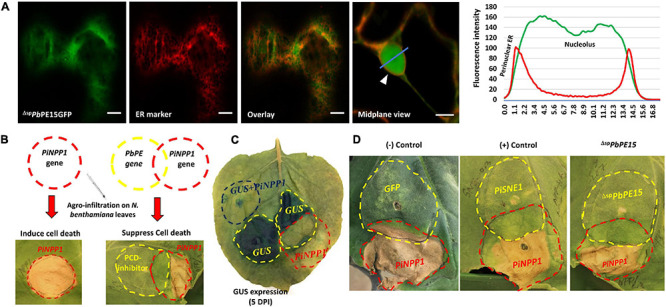
^Δ*sp*^*PbPE15* inhibits PCD induced by *PiNPP1*. **(A)** ^Δ*sp*^*Pb*PE15GFP co-localize with an ER marker (CD3-959) in *N. benthamiana* leaf epidermal cells. ^Δ*sp*^*Pb*PE15GFP shows nucleo-cytoplasmic localization with some ER fraction patterning after transient expression in *N. benthamiana* leaves. The surface view of the cell indicates cortical ER localization of ^Δ*sp*^*Pb*PE15GFP whereas, the mid-plane view of the merged images shows nuclear localization of the PE. White arrowheads indicate perinuclear ER. Fluorescence intensity plots on the right show the nuclear and perinuclear localization patterns of the FP-tagged proteins along the blue line on the image overlays. Scale bars = 10 μm. **(B)** Schematic of programmed cell death (PCD) suppression by *Agrobacterium* co-infiltration of PCD inducer (*PiNPP1*) and *P. brassicae PE* gene/cell death regulator. **(C)** Validation of the overlapping co-infiltration method for agro-infiltration with PCD inducers and *P. brassicae* PE gene/cell death regulators. **(D)** *P. brassicae* putative cell death inhibitor ^Δ*sp*^*PbPE15* results in inhibition of *PiNPP1*-induced PCD. An OD_600_ of 0.3 was used for all co-infiltration studies. The overlapping regions of the PCD test were identified as positive for the presence of necrosis or negative for the absence of necrosis. The *GFP* gene was used as a negative control whereas *PiSNE1* was used as a positive control. GUS = β-glucuronidase, *Pi*NPP1 = necrosis causing protein of *P. infestens*, *Pi*SNE1 = suppressor of necrosis 1 protein of *P. infestens*. The images are representative of three independent biological replicates.

*PiINF1*-induced PCD was significantly suppressed by *PbPE5*, *PbPE6*, *PbPE14*, and *PbPE15*, whereas co-expression of *PbPE13* and *PiINF1* resulted in an enhanced PCD response in *N. benthamiana* leaves (Figure 8C). *PbPE3*, *PbPE6*, *PbPE11*, *PbPE14*, and *PbPE15* significantly suppressed *PiNPP1*-triggered PCD (Figure 8D), with *PbPE15*, a putative member of the 2-oxoglutarate (2OG) and Fe (II)-dependent oxygenase superfamily, showing the strongest level of suppression of both inducers (Figures 8C,D). *Pb*PE15GFP shows nucleo-cytoplasmic localization with ER fractions after transient expression in *N. benthamiana* cells (Figure 9A). To validate PCD inhibition by *PbPE15*, an overlapping method of PCD inhibition, using the PCD inducer *PiNPP1* with *PbPE15*, was carried out (Figures 9B,D). The *P. infestans* suppressor of necrosis 1 (*Pi*SNE1), a secreted effector of the hemibiotrophic oomycete *P. infestans*, suppresses necrosis mediated by Nep-like proteins (NLPs) during the biotrophic infection phase and was used as a positive suppressor of cell death (Kelley et al., 2010).

*PbPE6* and *PbPE14* also showed significant suppression of both *PiINF1*- and *PiNPP1*-triggered PCD (Figures 8C,D). *PbPE2, PbPE10*, *PbPE12* and *PbPE17* had limited or no suppression effect on either *PiINF1*- or *PiNPP1*-triggered PCD (Figures 8C,D). *Pb*PEs localizing to the different compartments of the plant endomembrane system along with their significant regulatory roles in modulating PTI response indicate the functional importance of these effectors in plant pathogenesis during infection.

## Discussion

**FIGURE 10:**
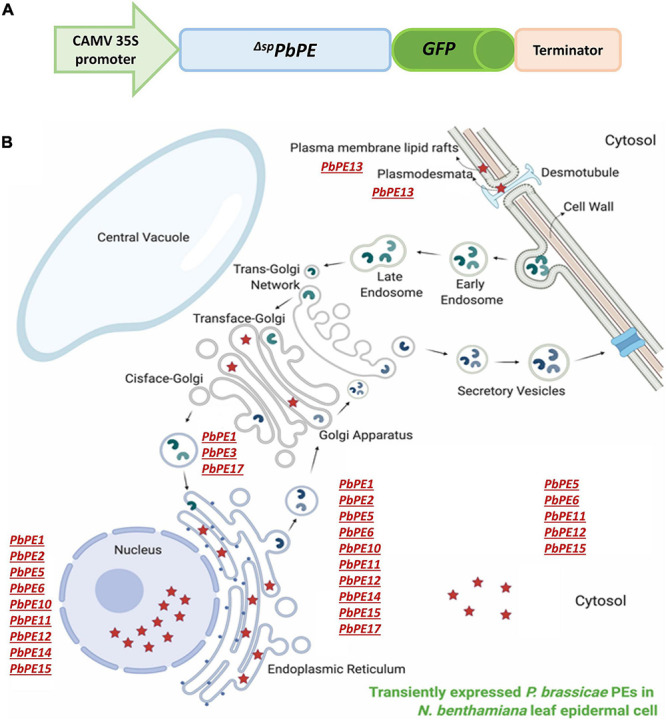
Schematics of the plant endomembrane localization of *P. brassicae* PEs after transient expression in *N. benthamiana* leaves. **(A)** Generation of P^*H*7*FWG*2^ vector-PE gene constructs for subcellular localization (not scaled). **(B)** Schematic of the localization of transiently expressed *Pb*PEs in distinct sub-compartments in a host cell. Red stars denote *P. brassicae* PEs.

A successful plant immune response against biotic stress requires a well-organized array of intracellular processes by the host, including signal transduction, endomembrane trafficking of cellular cargo to pathogen invasion sites in the process of the execution of PTI and ETI responses, resulting in some degree of resistance to the stress (Gu et al., 2017). As an intracellular biotroph, *P. brassicae* manipulates the host metabolism to its benefit while also avoiding recognition by host cells. In this study, we initially identified 52 *Pb*PEs from root galls of *P. brassicae* infected canola (*B. napus*), a subset of which were investigated further based on their localizations to the plant endomembrane system. Here, we report the impact of twelve endomembrane-targeting *Pb*PEs on cell death regulation in *N. benthamiana*. An overall schematic summarizing possible functions of the endomembrane-targeting *Pb*PEs during *P. brassicae* pathogenesis is presented (Figure 10).

The ER is the gateway to the cell’s secretory pathway, hosting the co-translational translocation of secretory proteins, protein folding, quality control (QC) system and ER stress response. The interconnected tubular network of the ER can extend throughout the cytoplasm from early endosome to PM, to cell-to-cell junctions, facilitating communication and the regulation of adaptive biotic stress responses. Due to both the receptive and the responsive nature of the ER (Breeze et al., 2020), it is a common subcellular target of pathogen effectors to manipulate host immunity and to hijack the secretory pathway to enable completion of the pathogen life cycle (McLellan et al., 2013; Jing et al., 2016; Fan et al., 2018; Meisrimler et al., 2019; Tsai et al., 2019). In oomycetes up to 17% of the effector secretome targets ER localized host proteins, e.g., ER-localized NAC transcription factors and ER luminal binding immunoglobulin proteins (BiPs; Khan et al., 2018a). ER proteins, such as the NAC transcription factors, are translocated to the nucleus to regulate gene expression, possibly carrying the pathogen effector protein with them into the nucleus (McLellan et al., 2013; Duan et al., 2017; Meisrimler et al., 2019).

Of the 12 endomembrane-targeting *Pb*PEs identified in this study, nine (*Pb*PE1, *Pb*PE2, *Pb*PE5, *Pb*PE6, *Pb*PE10, *Pb*PE11, *Pb*PE12, *Pb*PE14, and *Pb*PE15) showed ER, as well as nuclear localization in *N. benthamiana* leaf cells. Six of these effectors were found to suppress either *Pi*INF1 and/or *Pi*NPP1-triggered PCD. The most significant inhibition of PCD was observed with ^Δ*sp*^*Pb*PE15, a predicted member of the 2OG and Fe (II)-dependent oxygenase superfamily. A tobacco rattle virus (TRV)-based, host-induced gene silencing (HIGS) of 2OG-Fe(II) oxygenase compromised the pathogenesis of *Rhizoctonia solani* in tomato plants (Ghosh et al., 2020). Furthermore, treating *P. brassicae* infected *A. thaliana*, with an inhibitor of oxoglutaric acid-dependent dioxygenase, resulted in a decreased severity of clubroot formation (Päsold et al., 2013). As a predicted member of this superfamily, it would be appropriate for ^Δ*sp*^*Pb*PE15 to have a role in the processes leading to clubroot formation, including inhibitory effects on defense signaling in plants.

Several studies have previously shown that ER localization of pathogen effectors result in suppression of the pathogen-induced ER stress response and manipulation of trafficking routes to facilitate infection (Qiang et al., 2012; Jing et al., 2016; Fan et al., 2018). The RXLR effector, PcAvr3a12 from *Phytophthora capsici*, targets and inhibits a novel ER-localized plant peptidyl-prolyl cis-trans isomerase (PPIase), FKBP15-2, to facilitate infection by suppressing ER stress-mediated immunity (Fan et al., 2018). Similarly, the effector PsAvh262, from the soybean pathogen *Phytophthora sojae*, suppresses ER stress-triggered cell death and aids *P. sojae* infection by stabilizing plant ER luminal BiPs, thereby preventing them from participating in the ER-localized unfolded protein-related pro-survival response (Qiang et al., 2012; Jing et al., 2016). The inhibition of the ER-stress related defense mechanism can also result from the blocking of translocation of NAC transcription factors from the ER to the nucleus by RXLR effectors (McLellan et al., 2013; Meisrimler et al., 2019).

While *Pb*PE3 localized specifically to the plant Golgi bodies, suggesting a role in manipulating plant cell vesicle trafficking, as seen in Bartetzko et al. (2009), two *P. brassicae* ankyrin (ANK) repeat-containing proteins, ^Δ*sp*^*Pb*PE1GFP and ^Δ*sp*^*Pb*PE17GFP, localized to both ER and Golgi, with ^Δ*sp*^*Pb*PE1GFP additionally showing nuclear localization. ANK repeat-containing proteins, present in all eukaryotes and some prokaryotes (Mosavi et al., 2004), have diverse functions including roles in signal transduction, vesicular trafficking, disease resistance, reactive oxygen production, biotic and abiotic stress responses, cell cycle regulation, and control of gene transcription (Mou et al., 2013; Böttner et al., 2009; Li and Chye, 2004; Sakamoto et al., 2008; Yang et al., 2008; Ge and Shao, 2011; Yuan et al., 2013; Sharma and Pandey, 2016). ANK domains form molecular scaffolds for protein-protein interactions and in some intracellular pathogens, ANK repeat-containing proteins are essential virulence factors, for example in animals, the *Legionella pneumophila* AnkX protein prevents the fusion of the *L. pneumophila*-containing vacuole with late endosomes in infected macrophages as well as interfering with microtubule-dependent transport of ER-derived vesicles (Pan et al., 2008). A number of ANK repeat-containing effector proteins have been identified in the *P. brassicae* transcriptome, being expressed in both the primary and secondary infection stages in host plants (Chen et al., 2019; Pérez-López et al., 2020).

In a plant–pathogen interaction, the PM is one of the first barriers to infection by many pathogens. Located within the PM are distinct pattern recognition receptors (PRRs) that recognize microbial patterns and induce PTI responses to terminate or contain an infection (Hammond-Kosack and Jones, 1996; Jones and Dangl, 2006). PRRs are often located in cholesterol-rich lipid microdomains or lipid rafts, dynamically assembled and disassembled within the PM (Vieira et al., 2010) and targeted by some protozoan pathogens that have developed methods to escape PRR recognition (Mañes et al., 2003; Hartlova et al., 2010). *Plasmodium falciparum*, the causative pathogen for malaria, enters targeted cells via lipid rafts, whereas depleting lipid rafts of their cholesterol content was found to prevent infection (Lauer et al., 2000; Samuel et al., 2001). Other PM or ER-localized proteins that reside in close proximity of plasmodesmata, may prove to be targets of *P. brassicae* effectors to enhance pathogen cell to cell movement (Ueki and Citovsky, 2014; Sun et al., 2019).

Interestingly, ^Δ*sp*^*Pb*PE13GFP, highly expressed during the secondary infection stage of *P. brassicae* infection, localized to PM lipid rafts associated with plasmodesmata (PD). Significant lipid enrichment in the raft arrangement around PD, compared with the neighboring PM, providing a perfect medium for immune signaling and cell to cell communication by many glycosylphosphatidylinositol (GPI)-anchored and PD-localized proteins, has previously been reported (Malinsky et al., 2013; Grison et al., 2015; Sager and Lee, 2018; Sasaki et al., 2018; Jaillais and Ott, 2020). The lipid raft marker Remorin has been detected in association with PD, where they are thought to modulate the PD size exclusion limit (SEL) and regulate pathogen movement across the raft enriched PM (Raffaele et al., 2009; Perraki et al., 2014; Gronnier et al., 2017; Sasaki et al., 2018). In *N. benthamiana*, the *Pseudomonas syringae* effector HopZ1a, which interferes with early immune signaling at the PM in plants, interacts with the lipid raft localized protein REMORIN4 (*Nb*REM4) (Albers et al., 2019). Similarly in Arabidopsis, the *P. syringae* effector HopF2, which suppresses the PTI response by interacting with the BAK1 receptor at the PM, is also associated with the remorins *At*REM1.2 and *At*REM1.3 (Zhou et al., 2014; Khan et al., 2018b). Recently, cell to cell movement of *P. brassicae* plasmodial structures, as tracked by *PbBMST* movement, has been reported during infection (Badstöber et al., 2020). The association of ^Δ*sp*^*Pb*PE13GFP with remorins linked with PD suggests a role in pathogen spread, possibly through regulation of the plant PTI response. The compartmentalization of ^Δ*sp*^*Pb*PE13GFP fluorescence at the PM remorin-PD nanodomains, after flg22 treatment, supports this suggestion. flg22 interacts with Flagellin Sensing-2 (FLS-2), associated with lipid rafts and triggers the endocytosis of the FLS2-flg22 complex into endosomes that are sorted at the trans-Golgi network and targeted for degradation, probably in the vacuole (Jelenska et al., 2017). A similar pattern of fluorescence distribution, due to membrane raft reorganization, has been observed for GFP-*Nb*REM4 and FLS2, after flg22 treatment (Keinath et al., 2010; Albers et al., 2019). An increase in *B. rapa* FLS2 (BraA09g021780.3C) transcript levels has been observed after infection with a virulent strain (SCCD-52) of *P. brassicae* (Fu et al., 2019). The association of ^Δ*sp*^*Pb*PE13GFP with this complex suggests that it may have a role in hijacking the endocytosis response and redirecting the process toward a root-wide distribution of the pathogen.

None of the *Pb*PEs, in this study, contain HDEL/KDEL ER retention signals. As such, the localization of these *Pb*PEs to the endomembrane system would be the result of yet to be identified self-contained signals or host-mediated modification such as lipidation via *S*−palmitoylation, *N*−myristoylation or prenylation, previously reported for correct subcellular localization, membrane association and translocation from one cellular compartment to another, of pathogen effectors (Hicks and Galán, 2013; Escoll et al., 2016; Popa et al., 2016). Likewise, none of the *Pb*PEs that also localized to the nucleus (nine of the 12) contain any recognized nuclear localization signals (NLSs). While it is possible that nuclear localization resulted from the overexpression and diffusion of *Pb*PE-GFP to the nucleus, as was seen for GFP alone (Figure 2B; Wang and Brattain, 2007), it is probable that the *Pb*PEs were either targeted through yet to be identified self-contained signals (Savada and Bonham-Smith, 2013; Bourgeois et al., 2020; Tessier et al., 2020) or, due to the contiguous nature of the ER with the perinuclear membrane, localization resulted from the translocation or diffusion of *Pb*PE-GFPs from the ER lumen into the nucleus.

All of these studies were carried out in a heterologous (tobacco) system and we have to recognize that *Pb*PE localization could be a result of the system, as well, studying effector function in isolation may not reflect the true function when studied in combination with other effectors during a natural and spontaneous *P. brassicae* infection in host plants. Identifying *P. brassicae* PEs and deciphering their roles in the regulation of plant immunity during disease development will be important for understanding plant–pathogen interactions during clubroot establishment and the design of effective control strategies against this devastating pathogen.

## Data Availability Statement

The original contributions presented in the study are included in the article/ Supplementary Material, further inquiries can be directed to the corresponding author.

## Author Contributions

MH, EP-L, CT, YW, and PB-S designed the research, and wrote and edited the manuscript. MH performed the research. All authors contributed to the article and approved the submitted version.

## Conflict of Interest

The authors declare that the research was conducted in the absence of any commercial or financial relationships that could be construed as a potential conflict of interest.

## Footnotes

1 https://www.ebi.ac.uk/Tools/msa/muscle/

2 http://weizhongli-lab.org/cd-hit/

3 https://www.expasy.org/tools/

4 https://www.genscript.com/sms2/orf_find.html

5 http://www.cbs.dtu.dk/services/SignalP-5.0/

6 http://www.cbs.dtu.dk/services/TMHMM/

7 https://www.ebi.ac.uk/Tools/pfa/phobius/

8 https://prosite.expasy.org/scanprosite/

9 https://www.ebi.ac.uk/Tools/hmmer/

10 http://ekhidna2.biocenter.helsinki.fi/sanspanz/

11 https://www.ncbi.nlm.nih.gov/Structure/cdd/wrpsb.cgi

12 https://web.expasy.org/protparam/

13 http://localizer.csiro.au/

14 https://www.fishersci.nl/shop/products/software-vision-works-ls-complete-software/12568355

15 https://abrc.osu.edu/

16 https://imagej.net/Fiji

## Supplementary Material

Supplementary Figure 1 Identification and selection of *P. brassicae* putative effectors (*Pb*PEs) from *B. napus* clubroot gall cDNA library. Bioinformatics tools and parameters used in a signal peptide prediction pipeline to identify PEs of *P. brassicae*. **(A)** Prediction of *P. brassicae* secretome – cDNA sequences from a 35 DPI canola gall cDNA library were trimmed and filtered for the removal of duplicate sequences. Non-redundant sequence reads were then mapped against the *B. napus* and *P. brassicae* genomes to identify pathogen specific sequences using Spliced Transcripts Alignment to a Reference (STAR: Dobin et al., 2013). *P. brassicae* secretome sequences were identified from a BlastX search against the *P. brassicae* non-redundant proteome and positive hits were then searched by SignalP 4.1, with a D-cut-off score above or equal to 0.5, for the presence of a signal peptide (SP). **(B)** Identification of *Pb*PE candidates – *P. brassicae* secretory sequence reads with transmembrane domain(s) and ER retention signals (HDEL, KDEL) were removed from the list. The final group of *Pb*PE candidates was identified after signal peptide prediction using SignalP 5.0 (http://www.cbs.dtu.dk/services/SignalP-5.0/) with a D-cut-off score above or equal to 0.7. “*n*” denotes the number of reads belonging to a specific group in the pipeline. Encircled numbers specify a step in the pipeline and its detailed information. DPI = days post inoculation, STAR = spliced transcripts alignment to a reference, TMD = transmembrane domain, ER = endoplasmic reticulum.

Supplementary Figure 2 Nucleo-cytoplasmic localization with ER fractions of ^Δ*sp*^*Pb*PE5GFP, ^Δ*sp*^*Pb*PE6GFP, ^Δ*sp*^*Pb*PE11GFP, and ^Δ*sp*^*Pb*PE12GFP in *N. benthamiana* leaf epidermal cells. The *Pb*PEs show nucleo-cytoplasmic localization with some accumulation and distribution in the ER. Co-localization of ^Δ*sp*^*Pb*PE5GFP, ^Δ*sp*^*Pb*PE6GFP, ^Δ*sp*^*Pb*PE11GFP and ^Δ*sp*^*Pb*PE12GFP with the ER marker (CD3-959) in *N. benthamiana* leaf epidermal cells. Confocal images show nuclear localization of the effectors excluding nucleolar localization. Surface views of the cell show cortical ER association of the effector in *N. benthamiana*. White arrowheads indicate punctate structures of *Pb*PE localization. Scale bars = 10 μm.

Supplementary Table 1 List of *Plasmodiophora brassicae* effectors and their functional details. All the predictions and calculations done with the *Pb*PE sequences using different web servers are mentioned in the materials and methods. ¥ denotes GenBank accession number and the number range in bracket indicates nucleotide range of the protein coding sequence (CDS). Domain information of the proteins were identified using conserved domain database (CDD) search. C-site = AA = amino acid, Ch = chloroplast, cleavage site, mito = mitochondria, MW = molecular weight, NA = not available, nuc = nucleus, nt = nucleotide, and pI = isoelectric point.

Supplementary Table 2 List of plasmid constructs used in this study.

Supplementary File 1 Time-lapse of ^Δ*sp*^*Pb*PE17GFP colocalization with ER marker. Red fluorescence corresponding to the ER marker, mCherry-HDEL (CD3-959) shows the cortical ER pattern and the yellow signal indicates the merge of green and red fluorescence channels.

Supplementary File 2 Time-lapse of ^Δ*sp*^*Pb*PE17GFP colocalization with Golgi marker. Yellow fluorescence indicates the merge of punctate green fluorescence from ^Δ*sp*^*Pb*PE17GFP co-localizes with the cis-faced Golgi stack marker, *Gm*Man^11–49*aa*^-mCherry (CD3-968) in plants.
