# Supplementary figures and images for: Endomembrane-Targeting Plasmodiophora brassicae Effectors Modulate PAMP Triggered Immune Responses in Plants
Source: Front Microbiol. 2021 Jul 1;12:651279. doi: 10.3389/fmicb.2021.651279 (PMC8282356; doi:10.3389/fmicb.2021.651279)

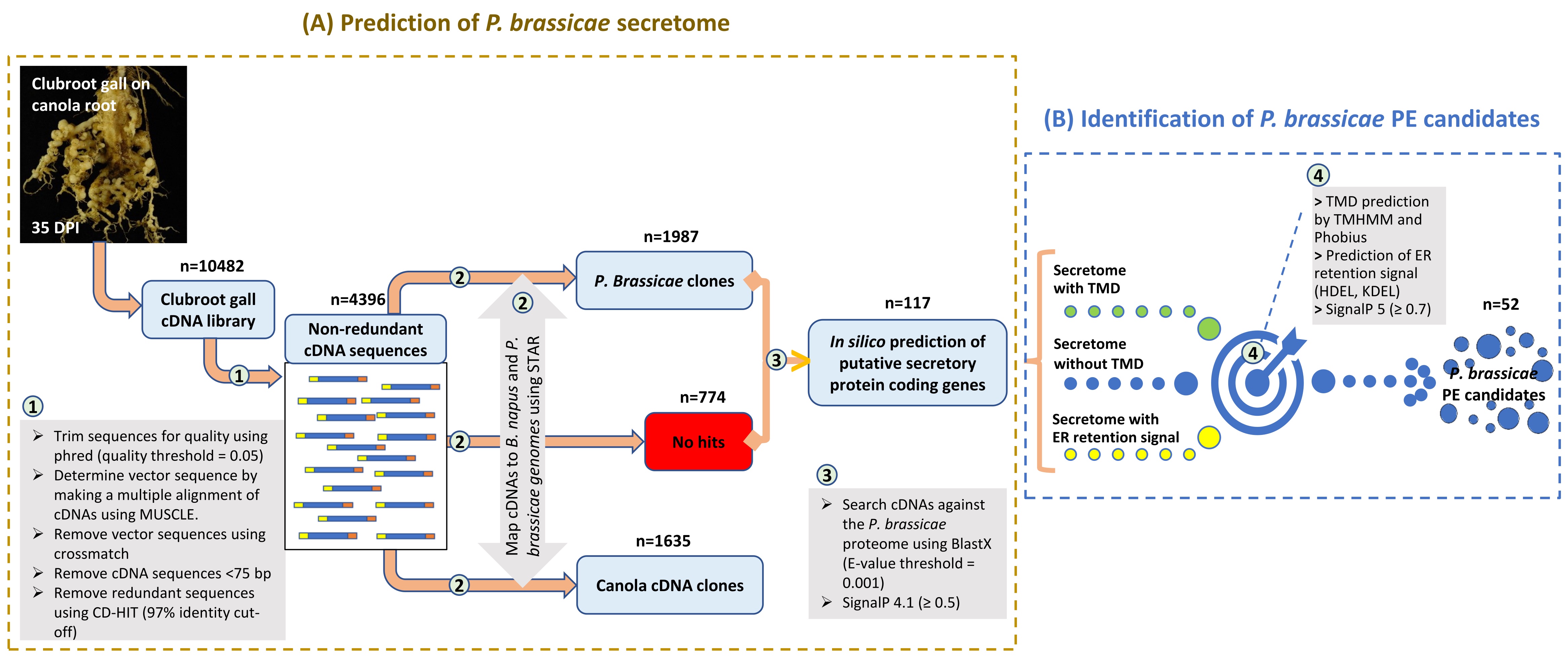

Supplement: Supplementary Figure 1 — Identification and selection of P. brassicae putative effectors (PbPEs) from B. napus clubroot gall cDNA library. Bioinformatics tools and parameters used in a signal peptide prediction pipeline to identify PEs of P. brassicae. (A) Prediction of P. brassicae secretome – cDNA sequences from a 35 DPI canola gall cDNA library were trimmed and filtered for the removal of duplicate sequences. Non-redundant sequence reads were then mapped against the B. napus and P. brassicae genomes to identify pathogen specific sequences using Spliced Transcripts Alignment to a Reference (STAR: Dobin et al., 2013). P. brassicae secretome sequences were identified from a BlastX search against the P. brassicae non-redundant proteome and positive hits were then searched by SignalP 4.1, with a D-cut-off score above or equal to 0.5, for the presence of a signal peptide (SP). (B) Identification of PbPE candidates – P. brassicae secretory sequence reads with transmembrane domain(s) and ER retention signals (HDEL, KDEL) were removed from the list. The final group of PbPE candidates was identified after signal peptide prediction using SignalP 5.0 (http://www.cbs.dtu.dk/services/SignalP-5.0/) with a D-cut-off score above or equal to 0.7. “n” denotes the number of reads belonging to a specific group in the pipeline. Encircled numbers specify a step in the pipeline and its detailed information. DPI = days post inoculation, STAR = spliced transcripts alignment to a reference, TMD = transmembrane domain, ER = endoplasmic reticulum. [file Image_1.JPEG]

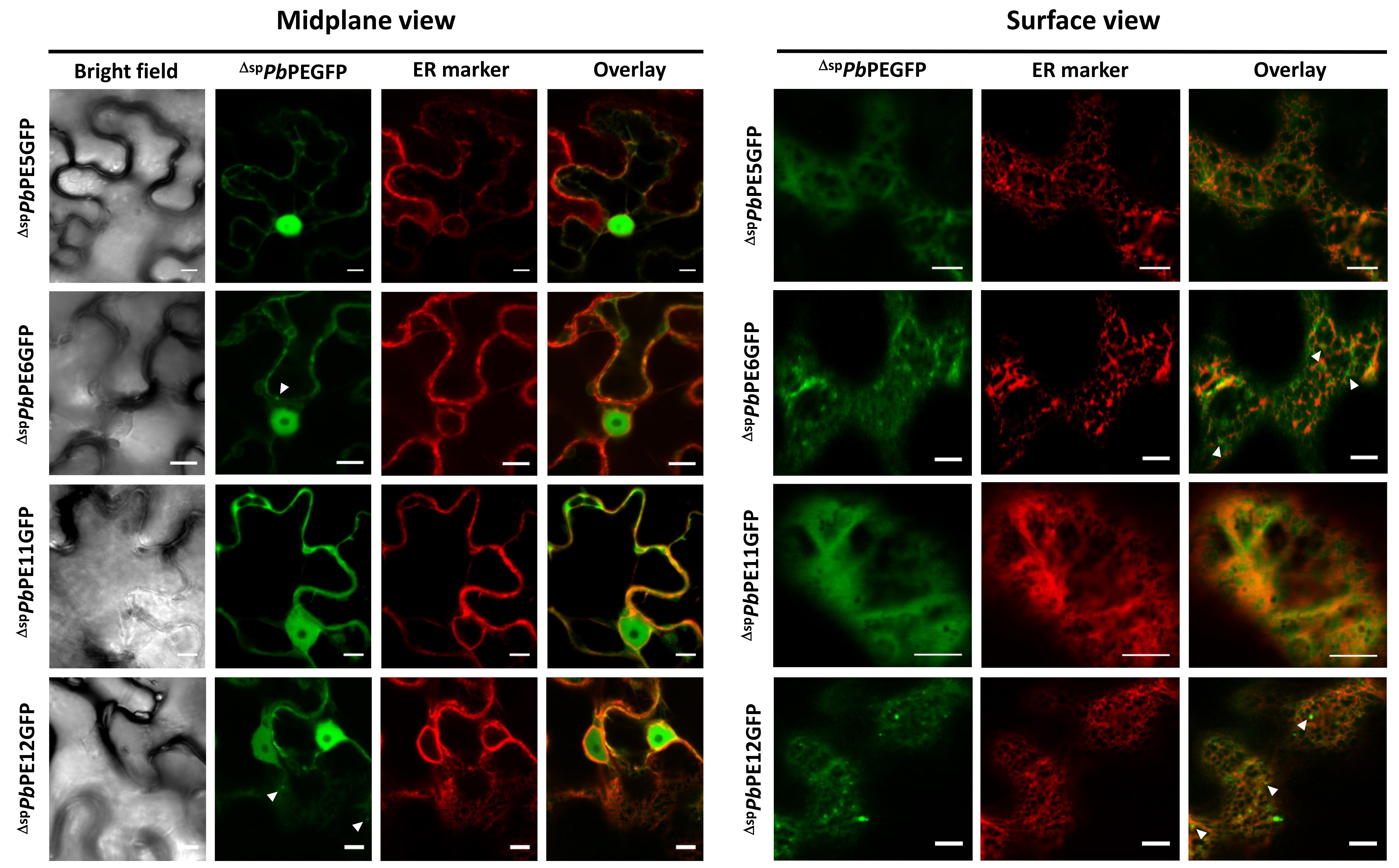

Supplement: Supplementary Figure 2 — Nucleo-cytoplasmic localization with ER fractions of ΔspPbPE5GFP, ΔspPbPE6GFP, ΔspPbPE11GFP, and ΔspPbPE12GFP in N. benthamiana leaf epidermal cells. The PbPEs show nucleo-cytoplasmic localization with some accumulation and distribution in the ER. Co-localization of ΔspPbPE5GFP, ΔspPbPE6GFP, ΔspPbPE11GFP and ΔspPbPE12GFP with the ER marker (CD3-959) in N. benthamiana leaf epidermal cells. Confocal images show nuclear localization of the effectors excluding nucleolar localization. Surface views of the cell show cortical ER association of the effector in N. benthamiana. White arrowheads indicate punctate structures of PbPE localization. Scale bars = 10 μm. [file Image_2.JPEG]
