# Supplementary material for: Endomembrane-Targeting Plasmodiophora brassicae Effectors Modulate PAMP Triggered Immune Responses in Plants
Source: Front Microbiol. 2021 Jul 1;12:651279. doi: 10.3389/fmicb.2021.651279 (PMC8282356; doi:10.3389/fmicb.2021.651279)
Supplement: Supplementary Table 1 — List of Plasmodiophora brassicae effectors and their functional details. All the predictions and calculations done with the PbPE sequences using different web servers are mentioned in the materials and methods. ¥ denotes GenBank accession number and the number range in bracket indicates nucleotide range of the protein coding sequence (CDS). Domain information of the proteins were identified using conserved domain database (CDD) search. C-site = AA = amino acid, Ch = chloroplast, cleavage site, mito = mitochondria, MW = molecular weight, NA = not available, nuc = nucleus, nt = nucleotide, and pI = isoelectric point. [file Table_1.DOCX]

**Table S1: List of *Plasmodiophora brassicae* effectors and their functional details.**

| ***Pb*PEs** | **Accession number**  **(*P. brassicae* pathotype 3 homolog)** | **Domain information** | **SignalP 5.0** | **C-site** | **AA** | **MW** | **pI** | **Cysteine- content** | **Location** | **Comments** |
| --- | --- | --- | --- | --- | --- | --- | --- | --- | --- | --- |
| *Pb*PE1 | PBRA_008871 | cd00204 ankyrin repeats | 0.919 | 26 | 407 | 44.9 | 7.0 | 6 (1.5 %) | NA |  |
| *Pb*PE2 | PBRA_003503 | NA | 0.997 | 26 | 283 | 30 | 4.5 | 10 (3.5%) | NA |  |
| *Pb*PE3 | PBRA_006110 | NA | 0.961 | 19 | 487 | 51.9 | 4.8 | 4 (0.8%) | NA |  |
| *Pb*PE4 | PBRA_000619 | NA | 0.994 | 20 | 145 | 15.6 | 11.1 | 1 (0.7%) | Mito | Chen *et al*., 2019 |
| *Pb*PE5 | PBRA_001722 | cd00204 ankyrin repeats | 0.981 | 31 | 389 | 42.4 | 7.3 | 2 (0.5%) | NA | Perez-Lopez *et al*., 2020 |
| *Pb*PE6 | PBRA_006553 | cd00180 Catalytic domain of Protein Kinases | 0.977 | 23 | 311 | 35.4 | 9.1 | 5 (1.6%) | Ch, Mito |  |
| *Pb*PE7 | PBRA_006345 | cd05488 Fungal Proteinase A | 0.971 | 22 | 395 | 43.7 | 6.1 | 5 (1.3%) | NA |  |
| *Pb*PE8 | PBRA_005609 | NA | 0.997 | 18 | 411 | 46.3 | 5.2 | 4 (1.0%) | NA |  |
| *Pb*PE9 | PBRA_005440 | NA | 0.888 | 18 | 266 | 27.7 | 4.7 | 0 (0%) | Nuc | Chen *et al*., 2019; Perez-Lopez *et al*., 2020 |
| *Pb*PE10 | PBRA_007744 | NA | 0.993 | 17 | 179 | 19.1 | 9.2 | 7 (3.9%) | NA |  |
| *Pb*PE11 | PBRA_007435 | cl17238 RING_Ubox super family domain | 0.99 | 21 | 251 | 27.3 | 5.1 | 8 (3.2%) | NA |  |
| *Pb*PE12 | SPR00520.1^¥^ | cl17238 RING_Ubox super family domain | 0.967 | 34 | 253 | 27.6 | 8.1 | 9 (3.6) | NA |  |
| *Pb*PE13 | CDSF01000098.1^¥^  (88873-89304) nt | NA | 0.993 | 17 | 143 | 16 | 9.1 | 2 (1.4%) | Ch |  |
| *Pb*PE14 | PBRA_002947 | cl07422 Sep15/SelM redox domain | 0.996 | 17 | 149 | 16.1 | 4.5 | 8 (5.4%) | NA |  |
| *Pb*PE15 | PBRA_003835 | cl21496 2OG-FeII_Oxy super family | 0.886 | 22 | 259 | 28.7 | 8.8 | 3 (1.2%) | Mito |  |
| *Pb*PE16 | PBRA_004763 | NA | 0.925 | 22 | 194 | 21.1 | 4.9 | 0 (0%) | NA | Chen *et al*., 2019 |
| *Pb*PE17 | PBRA_006690 | cl02518 BTB/POZ domain and pfam12796 Ankyrin repeats (3 copies) | 0.97 | 30 | 405 | 45.1 | 6.7 | 5 (1.2%) | NA |  |
| *Pb*PE18 | PBRA_008207 | NA | 0.914 | 24 | 140 | 14.6 | 4.7 | 2 (1.4%) | NA | Perez-Lopez *et al*., 2020 |
| *Pb*PE19 | PBRA_005421 | NA | 0.963 | 20 | 224 | 23.3 | 9.3 | 5 (3%) | Ch, Mito |  |
| *Pb*PE20 | PBRA_001818 | NA | 0.989 | 23 | 355 | 37.7 | 8.7 | 10 (2.8%) | Ch |  |
| *Pb*PE21 | AFK13134.1 | cl04109 SAM dependent carboxyl methyltransferase | 0.978 | 21 | 377 | 42.5 | 5.1 | 5 (1.3%) | NA | Jutta Ludwig‐Müller *et al*., 2015; Bulman S *et al*., 2019 |
| *Pb*PE22 | PBRA_004903 | cl09109 Nuclear transport factor 2 (NTF2-like) superfamily | 0.934 | 19 | 323 | 35.6 | 7.6 | 7 (2.2%) | Nuc |  |
| *Pb*PE23 | PBRA_003317 | cl21453 Protein Kinases, catalytic domain | 0.98 | 24 | 358 | 39.8 | 7.1 | 12 (3.4%) | NA |  |
| *Pb*PE24 | PBRA_000644 | NA | 0.917 | 27 | 171 | 17.8 | 5.2 | 6 (3.5%) | NA |  |
| *Pb*PE25 | PBRA_008084 | NA | 0.987 | 22 | 215 | 22.6 | 6.2 | 6 (2.8%) | NA |  |
| *Pb*PE26 | PBRA_008204 | NA | 0.983 | 17 | 162 | 16.5 | 5.2 | 2 (1.2%) | Ch |  |
| *Pb*PE27 | SGFA01000035.1^¥^  (555032-555314) nt | NA | 0.964 | 20 | 122 | 13.5 | 5.2 | 3 (2.5%) | NA |  |
| *Pb*PE28 | PBRA_000671 | NA | 0.848 | 17 | 215 | 22.9 | 9.5 | 3 (1.4%) | Ch |  |
| *Pb*PE29 | PBRA_001067 | cl21453 Protein Kinases, catalytic domain | 0.995 | 25 | 359 | 39.3 | 8.2 | 14 (3.9%) | Nuc | Perez-Lopez *et al*., 2020 |
| *Pb*PE30 | PBRA_008932 | NA | 0.899 | 28 | 240 | 27.5 | 6.8 | 6 (2.5%) | NA |  |
| *Pb*PE31 | PBRA_007749 | cl38908 BTB/POZ domain and pfam12796 Ankyrin repeats (3 copies) | 0.74 | 22 | 301 | 33 | 5.6 | 5 (1.7%) | NA |  |
| *Pb*PE32 | PBRA_004950 | NA | 0.791 | 19 | 433 | 46.9 | 4.3 | 0 (0%) | NA |  |
| *Pb*PE33 | PBRA_003151 | COG0484 DnaJ-class molecular chaperone with C-terminal Zn finger domain | 0.845 | 25 | 373 | 41.3 | 8.0 | 8 (2.1%) | NA |  |
| *Pb*PE34 | PBRA_007015 | pfam12796 Ankyrin repeats (3 copies) and pfam13637 Ankyrin repeats (many copies) | 0.819 | 28 | 348 | 38.2 | 5.6 | 5 (1.4%) | NA |  |
| *Pb*PE35 | PBRA_005624 | NA | 0.992 | 18 | 414 | 46.4 | 5.8 | 9 (2.2%) | NA |  |
| *Pb*PE36 | PBRA_007709 | NA | 0.832 | 17 | 173 | 18.4 | 11.3 | 2 (1.4%) | Mito |  |
| *Pb*PE37 | PBRA_008194 | NA | 0.928 | 29 | 131 | 13.6 | 5.7 | 3 (2.3%) | NA |  |
| *Pb*PE38 | SGFC01000084.1^¥^  (138823-139482) nt | NA | 0.942 | 24 | 219 | 24.2 | 6.4 | 10 (4.6%) | NA |  |
| *Pb*PE39 | PBRA_003987 | cd11010 S1/P1 nucleases and related enzymes | 0.962 | 17 | 290 | 32.2 | 6.0 | 8 (2.8%) | Nuc |  |
| *Pb*PE40 | PBRA_005367 | smart00220 Serine/Threonine protein kinases | 0.849 | 25 | 459 | 50.7 | 6.3 | 8 (1.7%) | NA |  |
| *Pb*PE41 | PBRA_004539 | NA | 0.986 | 21 | 348 | 38.2 | 5.6 | 0 (0%) | Nuc |  |
| *Pb*PE42 | PBRA_006026 | cd02248: Peptidase C1A subfamily and smart00848: Cathepsin pro-peptide inhibitor domain (I29) | 0.803 | 21 | 340 | 37.4 | 4.9 | 11 (3.2%) | NA |  |
| *Pb*PE43 | PBRA_007566 | NA | 0.909 | 16 | 458 | 50.7 | 9.1 | 10 (2.2%) | NA |  |
| *Pb*PE44 | PBRA_005133 | NA | 0.748 | 24 | 544 | 61.3 | 8.7 | 9 (1.7%) | Nuc, Mito |  |
| *Pb*PE45 | PBRA_007709 | NA | 0.832 | 17 | 173 | 18.4 | 11.3 | 2 (1.2%) | Mito |  |
| *Pb*PE46 | PBRA_007344 | NA | 0.99 | 22 | 396 | 42.3 | 8.8 | 5 (1.3%) | Ch, Mito, Nuc | Perez-Lopez *et al*., 2020 |
| *Pb*PE47 | PBRA_006119 | cd01838 Isoamyl-acetate hydrolyzing esterase-like proteins and GDSL-like Lipase/Acyl hydrolase family | 0.813 | 34 | 286 | 31.5 | 6.5 | 5 (1.7%) | Mito |  |
| *Pb*PE48 | PBRA_008733 | PRK05218 heat shock protein 90; Provisional | 0.989 | 25 | 785 | 88.6 | 4.8 | 2 (0.3%) | NA |  |
| PbPE49 | SGFA01000082.1^¥^  (105507- 106210) nt | NA | 0.989 | 17 | 461 | 51 | 5.9 | 7 (1.5) | NA |  |
| PbPE50 | PBRA_004344 | NA | 0.974 | 21 | 220 | 24.5 | 9.0 | 6 (2.7%) | NA | Chen *et al*., 2019 |
| PbPE51 | SGFA01000121.1^¥^  (496178-495834) nt | NA | 0.789 | 16 | 233 | 23 | 9.2 | 5 (2.1%) | Ch |  |
| *Pb*PE52 | PBRA_007868 | NA | 0.846 | 24 | 303 | 33.4 | 6.8 | 2 (0.7%) | Ch, Mito | Chen *et al*., 2019 |

Note: All the predictions and calculations done with the *Pb*PE sequences using different web servers are mentioned in the materials and method section. (¥) denotes GenBank accession number and the number range in bracket indicates nucleotide range of the protein coding sequence (CDS). Domain information of the proteins were identified using conserved domain database (CDD) search. nt=Nucleotide, C-site= Cleavage site, AA= Amino acid, MW= Molecular weight, pI= Isoelectric point, NA= Not available, Nuc= Nucleus, Ch= Chloroplast and Mito= Mitochondria.
