# Supplementary material for: Endomembrane-Targeting Plasmodiophora brassicae Effectors Modulate PAMP Triggered Immune Responses in Plants
Source: Front Microbiol. 2021 Jul 1;12:651279. doi: 10.3389/fmicb.2021.651279 (PMC8282356; doi:10.3389/fmicb.2021.651279)
Supplement: Supplementary Table 2 — List of plasmid constructs used in this study. [file Table_2.DOCX]

**Table S2: List of plasmid constructs used in this study**

| **Plasmid construct** | **Purpose** | **ABRC code** | **Reference** |
| --- | --- | --- | --- |
| pDONR™/Zeo | Donor vector, gateway cloning | NA | Invitrogen |
| pDONR207 |  | NA |  |
| pDONR™221 |  | NA |  |
| pH7WG2 | 35S promoter driven binary expression vector, gateway cloning | NA | Karimi *et al.,* 2002 |
| pH7**F**WG2 |  | NA |  |
| pH7**R**WG2 |  | NA |  |
| pH7WG2-*GFP* | GFP expression, experimental control | NA | This study |
| pH7WG2-*GUS* | GUS expression, experimental control | NA |  |
| pH7WG2-*PEs* | Transient expression for cell death assay and subcellular localization study | NA |  |
| pH7**F**WG2-*PEs* |  | NA |  |
| pH7**R**WG2-*AtREM1.3* |  | NA |  |
| pGR106-*PiNPP1.1* | Cell death assay | NA | Kind gift from Dr. Jocelyn K.C. Rose, Cornell University, USA.  Kelley *et al.,* 2010 |
| pGR106-*INF1* |  | NA |  |
| pART-*PiSNE1* |  | NA |  |
| pBIN20-mCherry-HDEL | ER-marker, co-localization study | [ABRC (CD3-959)](https://abrc.osu.edu/stocks/number/CD3-959) | Karimi *et al*., 2002 |
| pBIN20-*Gm*Man^11-49aa^-mCherry | Golgi marker, co-localization study | [ABRC (CD3-968)](https://abrc.osu.edu/stocks/number/CD3-968) |  |
| pBIN20*-At*PIP2A-mCherry | PM marker, co-localization study | [ABRC (CD3-1007)](https://abrc.osu.edu/stocks/number/CD3-1007) |  |
| *PDCB1*-DsRed2 | Plasmodesmata marker, co-localization study | NA | Yuan *et al.,* 2016 |
| pSUC2 | Signal peptide validation assay | NA | Oh *et al.,* 2009 |

Note: NA = not available.
